# Managing Eating Disorders Within Medicaid‐Funded Health Care Systems in California

**DOI:** 10.1002/eat.24320

**Published:** 2024-11-08

**Authors:** Erin C. Accurso, Jennifer Ling, Karen J. Mu, Noelle Bruton, Marta Perez, Ricki Wagner, Holly Snyder

**Affiliations:** ^1^ Department of Psychiatry & Behavioral Sciences University of California San Francisco California USA; ^2^ Philip R. Lee Institute for Health Policy Studies San Francisco California USA; ^3^ Alameda County Behavioral Health Department Oakland California USA; ^4^ San Francisco Department of Public Health, Behavioral Health Services San Francisco California USA; ^5^ San Mateo County Behavioral Health & Recovery Services, Youth Division San Mateo USA; ^6^ Solano County Behavioral Health Fairfield California USA

**Keywords:** eating disorders, health services, insurance, Medicaid, mental health services, mental health services administration, policy, publicly‐funded care

## Abstract

**Objective:**

This study describes the current management of patients with eating disorders (EDs) served by publicly–funded Medicaid behavioral health systems.

**Method:**

Behavioral health leaders across nine counties in California met on a quarterly basis to share experiences, challenges, and lessons in the management of EDs within publicly–funded service systems. Detailed notes were taken, and a qualitative content analysis was undertaken to identify key themes.

**Results:**

County leadership noted insufficient outpatient capacity and difficulty building capacity for ED treatment, in addition to extraordinary challenges when facilitating admission to out‐of‐network higher level of care programs, at significant expense. Several challenges were identified in building an internal ED workforce, including the fact that many providers weren't eager to treat EDs due to training burden, patient complexity, and high levels of clinician burnout. When a higher level of care was required due to lack of outpatient resources or patient symptom severity or complexity, leaders dedicated significant resources to identify and contract with an appropriate program and secure the necessary funds.

**Discussion:**

Our study supports the need for specialized ED treatment and case management, as well as standardized processes and centralized resources, in Medicaid‐managed care. Findings also indicate the importance of protecting against clinician burnout, possibly through reduced caseload expectations, financial incentives, or increased support. Future policy change could reduce administrative burden and clinician burnout by facilitating admission to and reimbursement for higher levels of care.

**Summary**

- Public health care systems face multifaceted challenges in providing eating disorders care.
- Behavioral health leaders within Medicaid‐funded systems noted insufficient outpatient capacity and difficulty building capacity for ED treatment, in addition to extraordinary challenges when facilitating admission to out‐of‐network higher level of care programs, at significant expense.
- Our study supports the need for specialized ED treatment and case management, as well as centralized resources, in Medicaid‐managed care.

Eating disorders (EDs) affect about 20% of the population (Galmiche et al. 2019), with the second highest mortality rate across psychiatric disorders (Arcelus et al. 2011). EDs are also associated with significant societal and economic costs, including those related to health care, lost individual and family productivity, lost wellbeing, and other societal economic costs (Deloitte Access Economics 2020; Streatfeild et al. 2021). Given high treatment costs as well as significant costs to the individual, their family, and society, it is critical to understand how EDs are managed in real‐world settings, especially for underserved populations. Minoritized populations have been underrepresented in ED research (Burke et al. 2020; Halbeisen, Brandt, and Paslakis 2022) even though ED rates are comparable to or even greater among individuals of color (Mikhail and Klump 2021; Rodgers, Berry, and Franko 2018) and those experiencing socioeconomic disadvantage (Carroll et al. 2024; Huryk, Drury, and Loeb 2021; Mikhail et al. 2021, 2023). Unfortunately, youth of color and those with public insurance experience significant disparities in access to eating disorders care (Accurso, Buckelew, and Snowden 2021; Moreno et al. 2023).

In particular, the Medicaid system faces a multitude of challenges that hinder the identification and treatment of youth with EDs, leaving a vulnerable population at high risk for poor outcomes (Accurso, Buckelew, and Snowden 2021; Crest et al. 2024). Recent research indicates that youth with EDs who have public insurance have relatively low outpatient therapy use but high rates of hospitalization (Mikhail et al. 2025). However, very little research has focused on EDs within the Medicaid‐insured population, despite the fact that Medicaid pays for most mental health treatment in the United States. In California, Medicaid (Medi‐Cal) provides services for approximately one‐third of the state (California Department of Health Care Services 2024a), including 5.5 million youth.

In the early 1990s in California, authority was transferred from the state to the counties for major safety‐net programs, including mental health, social service, and general health services. Consequently, each county is responsible for overseeing the organization, administration, and financing of mental health services for their residents. This decentralized system allows counties to have greater flexibility in responding to the local needs of their members and control how they allocate resources across various public services. However, it also leaves each individual county with the responsibility of managing complex health care systems without the efficiency of large‐scale centralized resources, with some counties being more disadvantaged based on their budget. County‐administered behavioral health plans administer specialty mental health services to Medicaid beneficiaries who meet specific criteria for medical necessity, as defined by severity of impairment, diagnoses, and history of trauma (California Department of Health Care Services 2021). Services must be rendered according to timely access standards (e.g., within 10 business days of request for non‐urgent outpatient treatment) (California Department of Health Care Services 2024b). The behavioral health plans can use a combination of county‐operated and sub‐contracted mental health providers to deliver outpatient and inpatient treatment services.

This study describes the current management of patients with EDs served by publicly–funded behavioral health systems in California, which vary widely in their organization and structure. Given documented gaps in publicly–funded care for youth with EDs, understanding more about the context of care is essential to identify challenges and inform targets for improvement. California's decentralized Medicaid system (Medi‐Cal) is particularly well‐suited to this investigation because service environments are diverse and likely reflect variability in Medicaid‐managed care across states.

## 1 Methods

The California Association of Health Plans initiated a series of meetings in November 2016 about EDs. In response, leaders across a smaller group of California counties met to exchange information about how ED services for youth are coordinated within each county, following completion of the California Association of Health Plans meetings. The first meeting was organized spontaneously at the initiative of one county leader who had informally connected with several other county leaders around issues related to ED care coordination. At this initial meeting of administrators across nine counties in 2021, the convened group agreed to continue meeting on a quarterly basis to support each other around the management of ED cases. Most of the counties were located in central California (San Francisco Bay Area, Northern San Joaquin Valley), with one county located in southern California (Inland Empire), representing exclusively metropolitan areas (medium metro, large central metro, and large fringe metro) (Ingram and Franco 2014) with poverty rates between 13% and 23% (*M* = 16.1), which very roughly reflects the general poverty range across the state (Bohn, Danielson, and Thorman 2019). Participating stakeholders (*N* = 32) included program specialists, evidence‐based practice coordinators, program chiefs, division directors, quality improvement specialists, medical directors, education/training directors, critical care managers, access line supervisors/managers, program supervisors, behavioral health managers/supervisors, utilization management directors, clinical review specialists, clinical services managers, and ED referral coordinators within child and young adult systems of care. The first author took detailed notes at each of eight quarterly virtual meetings (November 2021–2023). The organizing leader also took meeting minutes that were shared following each meeting. Selected participants across counties (including all co‐authors) reviewed, verified, and contextualized or corrected these integrated notes to ensure their completeness and accuracy. Notes were then synthesized into several overarching themes based on a content analysis, which relied on a lower level of inference interpretation that focused on explicit descriptions of content. Content generated by specific counties was attributed to them through numerical identifiers in brackets (e.g., [1,3]), with each number representing one county. This qualitative study synthesizes shared expertise from quarterly meetings across a two‐year period, focusing on shared learning and practices. This study was classified as exempt by the Institutional Review Board at UCSF.

## 2 Results

While there were nine participating counties, only seven counties are represented in the analysis. The other two counties engaged in a primarily observational manner without directly sharing information or otherwise contributing to discussions. There were several key areas of focus across meetings. One major goal of meetings was for counties to share and learn from one another given poor clarity around procedures for managing complex disorders and a lack of resources. Leaders highlighted the need to expand outpatient mental health services for youth with EDs and the challenges in building capacity due to clinician disincentives in working with ED clients (e.g., complexity, workload, burnout). As a result of limited mental health and medical outpatient ED services, there was a greater need for higher level of care (HLOC). However, gaining client access to HLOC was complicated due to a lack of existing contracts between counties and private HLOC ED treatment facilities and limited support for negotiating contracts, resulting in a lengthy contracting process. Further, HLOC was very expensive, and counties bore most of the financial burden. Finally, HLOC (even when provided) was challenged by several factors, including travel to programs being a significant barrier to access, as well as experiences with programs lacking cultural responsiveness. Themes are detailed below.

### 2.1 Need for Outpatient Mental Health Service Expansion

Although individuals with EDs represented only a minority of cases, county leadership reported “a lot of need for ED services” and struggling to meet that need [1,3,6], largely due to the severity and urgency of ED cases presenting for care and the shortage of mental health clinicians who feel comfortable working with this population. Several counties had offered their clinicians opportunities for ED training largely focused on family‐based treatment (FBT) [1,2,4] given its strong evidence base and endorsement in clinical guidelines for adolescents with anorexia nervosa, bulimia nervosa, and to a lesser extent, binge eating disorder (Hilbert, Hoek, and Schmidt 2017). However, the number of clients presenting with EDs far exceeded clinician capacity with ED expertise or experience, both for providers who were internally employed and those with whom counties externally contracted [1,3,4,6]. This was especially challenging following onset of COVID‐19 pandemic given the increase in mental health concerns and demand for general youth mental health services being “at a maximum” [5]. However, demand for other mental health services (e.g., anxiety) was easier to meet when spread across the full mental health workforce, whereas demand for ED services was especially challenging because most providers felt EDs were outside their scope of practice.

Without a designated outpatient ED program, each county was “constantly putting out fires” (i.e., clinical leaders and administrators were devoting a significant amount of time and resources to identifying appropriate treatment options due to lack of immediate access to specialized care or a sufficient level of support for clients presenting with urgent medical and/or psychiatric symptoms, or rapidly decompensating) [4]. As a result, more youth were requiring HLOC admissions [5]. Administrators believed that both initial delays in ED diagnosis and subsequent delays in accessing outpatient ED treatment (due to limited outpatient ED treatment capacity) compounded symptom severity such that HLOC was more appropriate due to increasing client acuity [1,5,6]. Given difficulty building an internal workforce, one county referred most ED cases out to providers in private practice [5] but who were often at capacity, emphasizing the need to expand and diversify referral options. Unfortunately, annual rates of clinician turnover within county mental health systems are very high (30%–60%) (Mor Barak, Nissly, and Levin 2001), leading to loss of talent. However, even when trained therapists stayed within the county mental health system, their caseloads were “always full” with non‐ED cases [1,4–5] with no simple solutions to make space for ED clients [4,5]. Further, many providers weren't eager to treat EDs due to training burden, patient complexity, and high levels of clinician burnout [5].

During the meeting period, the State was also working on a new initiative (CalAIM) that aimed to build a more coordinated, person‐centered, and equitable health Medi‐Cal system, with special attention to strengthening behavioral health services and better integration between behavioral and physical health care. In preparation for the implementation of this initiative, one representative advocated with CalAIM's contractors to prioritize ED coverage and cost‐sharing given the gap in care with increasing demand and high cost of services, especially for HLOC [4]. One county emphasized how time is essential for young people with ED‐related medical and/or psychiatric complications, and that the State did not seem to understand how cumbersome and problematic the contracting and admissions process is for accessing HLOC [5]. Several counties were struggling with how to coordinate care between behavioral health and medical providers [5], how to minimize burden of ED cases on overstretched utilization management staff [1], and how manage ED cases with co‐occurring conditions [7]. Several representatives noted the importance of a team approach and how challenges arose in the absence of an integrated ED team [2,4,6]. Access to specialty medical care for EDs was variable, with some counties having access to a local academic medical center with specialty ED medical care [4,5] while others were working to establish partnerships with federally qualified health centers [1,6]. Another challenge to providing health care services was geographical distance, particularly for counties encompassing a large geographical area [5], with limited resources (e.g., two ED providers in total for the county), or further from academic tertiary care centers whose medical services could support mental health services. Additional details on ED management and outpatient care are available in Table 1, inclusive of problems identified, solutions implemented, and other lessons learned.

**TABLE 1:** Problems serving youth with eating disorders in the outpatient service system. [Correction added on 31 January 2025, after first online publication: Table 1 has been replaced.].

Problems	Efforts, goals, and lessons
**Access to outpatient mental health care**	
Building capacity	
*ED cases not being identified* Limited detection primary care and school settings, with behavioral health services often not initiated until discharge from a medical hospitalization [1,3] One county experienced continued growth in costs (including HLOC) as more EDs were identified [3]	*Counties had attempted to build an ED clinician panel through internal ED training of 5*–*10 providers* [1,4–5] Importance of regular “re‐training” [2,4] and including supervisors in training so that ED expertise is retained in the system when front‐line clinicians leave [4] Prioritizing on‐demand online training in EDs and family‐based treatment specifically Engaging in follow‐up support over time (four counties had a regular consultation group, typically monthly, with either an internal expert and/or external ED expert [1–2,4–5])
*Leaders wanted to expand ED services and commiserated about the difficulty of building an ED workforce* [1,4–6], *exacerbated by*:	
Prolonged hiring periods Low levels of ED experience in applicants given relatively limited ED education in most mental health training programs Poor access to training High rates of turnover, making internal expertise “obsolete” within 3–4 years “Infrequency and variability gap” (i.e., providers infrequently treat individuals with EDs, who have high variability in their treatment course), which makes it more difficult for providers to gain ED experience and retain their ED knowledge	
	*Other efforts to expand outpatient services*: Build an internal “dream team” of ED providers, including through potential use of Mental Health Services Act funds [7] or development of a Full‐Service Partnership^a^ for EDs [1,5–6]. Counties hoped that an ED‐specific Full‐Service Partnership team would be possible under CalAIM Enthusiasm and hopefulness expressed about a notice from the State indicating joint responsibility between Managed Care Health Plans and Mental Health Plans to cover medically necessary ED care, including PHP and residential treatment (California Department of Health Care Services 2022)
*EDs often require more intensive services than those typically provided in outpatient care*	*Focused on efforts that would facilitate higher intensity of service delivery*: Delivered ED training to agencies who provided FSP level of care Made an exception to automatically approve all ED cases for the FSP level of care whether or not they met formal criteria
Clinician time commitment and burnout	
Clinicians disincentivized to treat ED cases because of their complexity (often co‐occurring trauma) and time demands (e.g., 3 h/week instead of 1), which is not accounted for in clinician caseload Clinicians often “holding” severe ED cases who were awaiting admittance to a HLOC and spending more time on case management given the urgency of need (e.g., exploring access to HLOC programs, calling medical providers, consulting), leading to increased clinician stress and burden	Consider feasibility of clinician caseload taking into account time demands per case, rather than a set number of patients, so that clinicians are not disincentivized to treat ED cases [1,6] Clinicians appreciated opportunities to learn more about EDs and their treatment through training, and specialized services have expanded as a result In response to clinician burnout and overwhelm, one county developed an internal Eating Disorder Collaborative to mitigate feelings of professional isolation, facilitate collaboration among providers, and enhance the overall care system for EDs [5]
Geography	
In‐person office‐based services often incompatible with the goal of minimizing family transportation to access care	Given challenges with accessible transportation, fixed working schedules, and other caregiving demands [1], telehealth and in‐home services were critical to improving access to care
**Access to outpatient medical care**	
*Challenges with medical care included the following*:	
Poor access to medical care due to federally qualified health center closures and lack of ED expertise [6] Delays in access to medical care due to medical evaluations being provided only after initiating mental health care [5] Delays in access to HLOC given difficulty accessing outpatient medical care, leading some patients/families to go to the emergency department to submit required vitals for HLOC admission [5] Uncertainty about when to involve Child Protective Services due to potential medical neglect when caregivers were not following through with the recommended treatment [1,3]	
**Lack of clear procedures and resources for managing complex disorders**	
*County leadership often expressed uncertainty about how best to manage care for members with EDs, with a general lack of resources and supports to determine the best path forward*	There was high value placed in “keeping eating disorders on the radar,” learning from one another, and reducing isolation: “Misery loves company, and it's helpful to know that we're not the only ones with the monumental task of serving this population” [5] *Guidance was frequently sought on the following topics*: Placements for medical hospitalization Specific HLOC programs, including residential, partial hospitalization programs, and intensive outpatient programs, including soliciting recommendations for specific clients for whom an appropriate placement was unclear
	ED screening and assessment procedures Referral management Systems of care Contract negotiations Training opportunities
	*Some counties had developed processes around managing ED referrals, including the following*: Creation of a part‐time ED coordinator position (who was bilingual in English and Spanish given language need) [7] Development of a small ED committee that would evaluate clinical need for HLOC and support the treating clinician [4]
*Cases are complex to manage (due to the need for mental health and medical treatment) and require close coordination between providers on the treatment team*	*Several initiatives were instituted to support interdisciplinary care*: Establish regular meetings with interdisciplinary team members to facilitate coordination of services [2,6], including leveraging wraparound services, family partners Co‐location with pediatricians to reduce clinician isolation/burnout and facilitate coordination [2] Supplement care with additional interdisciplinary team members (e.g., hiring a part‐time dietitian to support EDs) [6] Integrate a caregiver peer to support treatment for families feeling overwhelmed, confused, and/or experiencing high levels of ambivalence about treatment [6]

^a^ Full‐Service Partnerships (FSPs) are the highest level of outpatient mental health services offered by California counties in a capitated model akin to intensive case management, providing comprehensive and intensive services that incorporate a team approach to behavioral health care for youth and their families.

### 2.2 Clinical Need for HLOC

Several counties had internal processes for determining level of care, generally including an in‐house provider and an external consultant or internal administrative leadership [1,4]. Another county had contracted out HLOC assessments to a third party because the difficulty in determining level of care and appropriate programs [6]. Counties regularly encountered challenges when supporting clients to access a HLOC. Several expressed a desire for their managed care plan to hold the contracts with HLOC programs, similar to how substance use disorder care is managed, which would avoid each individual county having to go through an arduous, lengthy contracting process and allow counties to reimburse the managed care plan [3,6]. Counties struggled with the fact that inpatient providers often recommended one or two specific HLOC programs, which were often not feasible due to contracting or being out‐of‐state.

A shared frustration across counties was that HLOC programs could not be billed directly to Medi‐Cal, requiring counties to pay for care upfront, often using general mental health funds [1,4–5]. Average treatment costs within a single program (e.g., residential placement for 6–8 weeks, or inpatient placement for 4 weeks) were approximately $100,000, and average treatment costs for a single member ranged from $200,000 to $300,000 [3]. Most counties had cost‐sharing agreements with their managed care payor, who would later reimburse a portion of their costs [2], but in some cases, their managed care plan wasn't sharing any of the cost [1]. Through discussion, counties realized that their negotiated cost‐sharing rates differed significantly from one another, even for the same program. Counties were hopeful that the state would provide some guidance on cost‐sharing [5].

County leadership reported feeling demoralized after dedicating significant resources and funds for a member to engage in HLOC, with clinical improvement confined to the duration of admission [1], symptoms that appeared worse upon discharge, or moving from one HLOC program to the next without benefit [3]. High rates of relapse were observed following discharge from residential programs, in part due to the fact that clients were discharged to home in the absence of accessible step‐down options [3,5], which would at times lead to a cycle of ineffective readmissions to HLOC [1,3,5], leading to a moral quandary of allocating limited resources towards a single gravely ill member, given high and multiplicative costs for HLOC admissions, especially when participation in these programs are not producing good long‐term outcomes [3].

Counties noted the psychiatric complexity and difficulty of ED cases (e.g., trauma history, language interpreter needs, socioeconomic or cultural barriers to treatment, treatment ambivalence), which they believed led to early discharge or patients leaving against medical advice from HLOC programs. Programs might discharge patients due to “non‐compliance” or “inability to benefit” from the program, with a recommendation for longer‐term care at a HLOC program that is not contracted with the county [1,3] or an out‐of‐state HLOC program, with whom counties cannot contract. Therefore, administrators end up “running around in circles” trying to identify programs that can meet a client's needs. Local HLOC programs are preferred for youth [7], but these are not always available due to long waitlists; adults are more often sent to southern CA for treatment due to more flexibility. Within the population of youth with EDs, HLOC was required for a minority of clients. However, youth with EDs required a HLOC significantly more often than youth with other psychiatric disorders. Further, care coordination needs for clients requiring a HLOC were significant, in large part due to the medical acuity of patients, administrative anxiety about patient risk, and variability in cost‐sharing agreements between mental health and medical plans. As a result, the demand on administrators to manage HLOC needs for ED clients far exceeded available resources and stood in stark contrast to the resources required for managing other psychiatric disorders. Further, resources used for “high‐intensity clients who cycle in and out of treatment for two years” limited resources for other members, often having done well in program but relapsing upon discharge to home [3–4]. Several counties were excited to have access to a virtual HLOC resource through a contract with their managed care plan. Additional details on HLOC are available in Table 2.

**TABLE 2:** Complications in accessing higher levels of care (HLOC) for youth with eating disorders. [Correction added on 31 January 2025, after first online publication: Table 2 has been replaced.].

Contracts
*Lack of contracts and lengthy contracting process* Once a HLOC program was identified, the first step was to determine whether a contract or single‐case agreement was feasible Many HLOC programs were unwilling to contract with counties (or unwilling to contract with additional counties) due to high administrative burden to become Medicaid compliant (e.g., required documentation, liability) and negotiate terms [6] Contracting process (single‐case agreements between the county and program) was laborious, often taking between 6 and 9 months (although there was significant variability depending on managed care plan), during which time the member would be waiting to receive an appropriate level of care Single‐case agreements were still sometimes needed, even when counties had several active contracts with HLOC programs, because availability was not guaranteed [4] *Lack of support in negotiating contracts* Managed care plans generally not taking the lead on identifying or contracting with HLOC programs, leaving the burden on the county Although advantageous to have the managed care payor establish the HLOC contract, one county noted the potential disadvantage of not receiving regular weekly updates on the member's care, attributed to the fact that they were not the entity with whom the program was contracted [6]
Financial considerations
*Providing HLOC to members with EDs was very expensive* One county allocated more than $500,000 of county general funds for ED services but often exceed the budget, usually for a couple of members cycling in and out of residential treatment [1] Some HLOC programs had different rates for different clients and across different counties, leading to an effort to negotiate a consistent rate across counties [3] *Managed care plans generally pay for the medical component of treatment but split the cost of the behavioral health component* Managed care plans take on greater financial responsibility for programs providing more medical care (e.g., 50/50 split for inpatient or PHP care, where both medical and psychiatric care are being provided) and less responsibility financial for programs providing primarily mental health care (e.g., 80/20 split for residential care) [3,7] Cost‐sharing rates for behavioral health varied from 0% to 50% One county lamented having accepted 20% cost‐sharing when learning that another county had negotiated for double that amount from the same managed care plan [3] Behavioral Health Information Notice 22–009 (California Department of Health Care Services 2022) addressed shared financial responsibility between managed care plans and counties, which counties hoped would facilitate HLOC admissions through ongoing agreements (instead of negotiations per placement, per child) Responsibility for payment for a HLOC was particularly unclear for carve‐outs (e.g., Kaiser Medi‐Cal)
Appropriateness
*HLOC programs often not equipped to provide culturally responsive care* Many HLOC programs perceived as being designed for more affluent families, or lacking in cultural responsiveness, sensitivity to families across socioeconomic levels, and/or poorly designed for clients with high complexity [6] Treatment model required a high level of client and caregiver engagement, but interpreters were not always available, and program materials were not available in languages other than English [3,7] Poorer engagement of caregivers speaking a language other than English when program staff do not have language capacity to accommodate the family, leading to poor outcomes even for relatively “straightforward” cases Given above challenges, identifying appropriate HLOC programs was very time‐consuming
Geographical location
*Proximity of HLOC programs was a significant barrier to accessing care* Available HLOC programs were often not available locally, possibly several counties away, and often too far for patients/families to travel [5,6] Transporting patients to a HLOC program was a significant burden (high transportation costs, lost family income due to time off work) to place on families who are struggling economically [5] Medi‐Cal does offer free transportation for medical appointments, but this service could be unreliable, it was not available to higher risk members, and several counties were not aware of this service Sometimes mental health support staff would drive clients from school to program, instead of the family Some members were required to relocate to access services (e.g., out‐of‐state residential treatment) [6] Counties agreed that they would benefit greatly from an “in‐house” HLOC program but lamented that this would not be feasible given inadequate demand [1] Telehealth programs were appealing to address geographical constraints and related burden placed on the family [6], but they were perceived as ineffective for many teens Clients engage in more ED behaviors when HLOC programs are virtual because they can more easily misrepresent what they are eating (during 1‐3 supported meals/snacks in program) or engage in movement that is not visible on camera Programs had technology requirements that were inaccessible for many Medi‐Cal members

## 3 Discussion

This study highlights the multifaceted challenges of organizing publicly–funded systems of care to care for young people with EDs. Findings underscore the urgent need for expanding outpatient mental health services for EDs, with current demand exceeding capacity, leading to an overreliance on HLOC. However, health care systems struggled to build and sustain internal expertise in EDs due to high clinician turnover rates and related high levels of burnout for clinicians working with ED clients, given the complexity and intensity of ED cases. The reliance on providers outside of the Medi‐Cal network, who are often at capacity, highlights a significant gap in the system that needs to be addressed through strategic workforce planning and service expansion. Financial and resource constraints also play a critical role in the challenges faced by counties, particularly given the complexities of contracting with HLOC programs, the financial burden of directly reimbursing HLOC programs for costly services (versus billing Medi‐Cal), and significant variability in negotiated cost‐sharing rates. Finally, differential access to specialty medical care led to additional inefficiencies in care. With limited (and finite) resources and in the absence of state‐level guidance or support on managing ED care, each county was left struggling with how best to allocate resources in a financially responsible, equitable, and ethical manner. As a result, leaders placed a high value on being in community with other leaders facing similar challenges and learning from others' experiences with screening and referral processes, systems of care, HLOC placements, managing negotiations with managed care payors, among other topics.

These challenges highlight the importance of flexible, on‐demand, recurring training to sustain ED expertise and ongoing consultation to support ED treatment implementation within counties. Nevertheless, training and re‐training to manage high rates of clinician turnover is inefficient and costly, particularly for highly complex disorders that require specialized training and coordinated, interdisciplinary management. One alternative solution would be to develop specialized ED teams that could centralize expertise and resources by providing care across counties (e.g., via telehealth, which may decrease barriers to accessing care). Retaining talent would likely be easier within a specialized program whose employees have demonstrated a commitment to ED care, but retention would be further bolstered through strong organizational leadership, augmented salary, and strong benefit packages. Otherwise, prior experience has demonstrated that a substantial number of FBT‐trained clinicians working in publicly–funded settings leave the public workforce (e.g., start private practice or join a private group practice) or stop providing direct clinical service (e.g., through promotion to a leadership role) (Borges et al. 2024).

Several counties experimented with a similar model by contracting with private specialized outpatient programs but reported relatively limited clinical success, citing barriers to treatment engagement for Medicaid‐insured youth and their families that required more nuanced and skilled treatment implementation. However, it is possible that building a specialized program with providers who have more local expertise of the strengths and challenges of Medicaid‐insured youth might be more effective, should such a model be feasible in the context of decentralized mental health care. However, significant efforts would be needed to protect clinicians against burnout, which is particularly high among mental health providers with demanding caseloads and those working with complex populations. Clinician burnout may decrease effectiveness, negatively impact patient outcomes, and lead to greater clinician turnover (Yang and Hayes 2020). Counties had already developed several solutions to increase support and protect against clinician burnout. For example, several counties agreed on the value of an ongoing consultation group with an ED expert to provide ongoing support for clinicians carrying ED cases. One county found that co‐location with pediatricians was effective in reducing clinician isolation and burnout, as well as facilitating care coordination, while other counties bolstered support by creating positions for new clinical staff (e.g., ED dietitian), admin support staff (e.g., ED care coordinator), and even caregiver peers. Creating new systems for managing ED care and increasing structure was another strategy implemented that likely reduced clinician (and administrator) burnout. Given the complexities of managing ED care, leaders found much benefit in creating additional systems to centralize and standardize processes to prevent continuous development of new processes. For example, one county had developed a committee to evaluate need for HLOC, while other counties had contracted this process out to a third party. Other counties had worked to establish regular meetings with interdisciplinary team members to facilitate coordination of services.

The findings also point to the importance of policy‐level interventions that might facilitate more transparent and standardized cost‐sharing arrangements with managed care payors, streamlined contracting processes when a HLOC is indicated. A prior notice in California recognized the importance of medically necessary ED care, including PHP and residential treatment (California Department of Health Care Services 2022), possibly foreshadowing future policy change that would support this need. If such a policy were instituted in California or any other state (allowing for PHP/residential treatment to be billed directly to Medicaid), it would allow for an investigation of its impact on direct and indirect health care‐related costs as well as patient outcomes. Further, policy changes may support incentivizing clinicians who are willing to engage in training and treat specialized, complex, and high acuity populations through reduced caseloads and/or greater salaries, with the rationale that increasing reimbursement for outpatient health care staffing would likely significantly reduce spending on HLOC programs (e.g., residential treatment, inpatient admissions). Within California, the upcoming implementation of the CalAIM initiative may shed light on systems‐level interventions that may improve care for the ED population. Future research may evaluate the impact of CalAIM on the provision of care, and other health systems may benefit from implementing initiatives with demonstrated effectiveness. Future research can also evaluate how the implementation of policy‐level changes may improve the administrative burden on counties for ED care coordination, quality of care, and costs, which could inform policy development or system improvements in other publicly–funded healthcare settings. Finally, these findings highlight the value of counties learning from each other's experiences, sharing expertise, and centralizing resources.

A key limitation of these findings is that findings are constrained to a group of counties within one state in the U.S. and the relative lack of variability across counties. While the counties were relatively diverse with respect to poverty level, they were all metropolitan and geographically close to one another, which may not generalize to nonmetropolitan counties. Although nonmetropolitan counties likely face similar (albeit possibly magnified) challenges, they also face several unique challenges not captured in this study, such as those related to geographical distance and limited access to specialized care, as well as more limited resources that are not as pronounced in urban areas. Further, the findings are restricted to Medicaid managed care in the state of California. The counties who participated in quarterly meetings were attuned to the need for eating disorders services and highly motivated to improve services for this population, which may not generalize to other health care systems. Finally, the organization of publicly–funded mental health services in the U.S. is quite different than in other countries, especially countries with national health care systems or those that directly finance mental health services across levels of care. Even so, some of the lessons around the potential utility of specialty teams and conditions necessary for protecting against burnout would apply across contexts.

In conclusion, while the study provides valuable insights into the need for expanded outpatient mental health services and the challenges faced by counties, it also highlights the necessity for targeted solutions. Future efforts should focus on developing tailored strategies to address the specific needs of different regions, improving coordination between behavioral and medical care, and establishing more consistent financial and contractual practices. Addressing these limitations and implementing comprehensive reforms will be essential in improving the overall effectiveness of mental health services and reducing the strain on HLOC programs.

## Author Contributions

**Erin C. Accurso:** conceptualization, data curation, formal analysis, funding acquisition, investigation, methodology, writing – original draft. **Jennifer Ling:** conceptualization, validation, writing – review and editing. **Karen J. Mu:** conceptualization, validation, writing – review and editing. **Noelle Bruton:** conceptualization, validation, writing – review and editing. **Marta Perez:** conceptualization, validation, writing – review and editing. **Ricki Wagner:** conceptualization, validation, writing – review and editing. **Holly Snyder:** conceptualization, validation, writing – review and editing.

## Ethics Statement

This study was classified as exempt by the Institutional Review Board at UCSF.

## Conflicts of Interest

Dr. Accurso has consulted with Partnership HealthPlan of California (a health care organization that contracts with the state to administer Medicaid benefits) concerning strategies to improve the treatment of eating disorders. The other authors have no conflicts to declare.

## Data Availability

The data that support the findings of this study are available directly from the author upon reasonable request.
